# Advances in large-scale DNA engineering with the CRISPR system

**DOI:** 10.1038/s12276-025-01530-0

**Published:** 2025-09-01

**Authors:** Lee Wha Gwon, Isabel Wen Badon, Youngjeon Lee, Ho-Joong Kim, Seung Hwan Lee

**Affiliations:** 1 National Primate Research Center, Korea Research Institute of Bioscience and Biotechnology, Cheongju, Republic of Korea; 2 KRIBB School of Bioscience, University of Science and Technology, Daejeon, Republic of Korea; 3 Department of Life Science, Chung-Ang University, Seoul 06974, Republic of Korea; 4 Department of Biology and Environmental Science, University of the Philippines Cebu, Cebu City, Philippines; 5 Department of Chemistry, Chosun University, Gwangju, Republic of Korea

**Keywords:** Gene therapy, Molecular engineering

## Abstract

In recent years, DNA engineering technology has undergone significant advancements, with clustered regularly interspaced short palindromic repeats (CRISPR)-based target-specific DNA insertion emerging as one of the most rapidly expanding and widely studied approaches. Traditional DNA insertion technologies employing recombinases typically involve introducing foreign DNA into genes in vivo by either pre-engineering recognition sequences specific to the recombinase or through genetic crossing to incorporate the requisite recognition sequence into the target gene. However, CRISPR-based gene insertion technologies have advanced to streamline this engineering process by combining the CRISPR–Cas module with recombinase enzymes. This process enables accurate and efficient one-step insertion of foreign DNA into the target gene in vivo. Here we provide an overview of the latest developments in CRISPR-based gene insertion technologies and discusses their potential future applications.

## CRISPR enhances targeted DNA insertion for genome engineering

As scientists work to understand complex traits and develop new therapies, they need tools to edit large sections of DNA. Traditional methods such as recombinases are constrained by their requirement for highly specific DNA sequence recognition, which limits their versatility and applicability. This study focuses on clustered regularly interspaced short palindromic repeats (CRISPR) systems, which use reprogrammed guide RNA for precise changes. However, CRISPR can create double-strand breaks, which can cause unintended changes. To address this, scientists are developing methods like prime editing, which makes precise edits without breaking both DNA strands. The study also highlights recent advances in CRISPR-based approaches for large-scale DNA integration. These methods show promise but need further refinement for use in human cells. Researchers conclude that while progress is being made, more work is needed to improve efficiency and reduce unintended effects.

This summary was initially drafted using artificial intelligence, then revised and fact-checked by the author.

## Introduction

As genomic research advances toward understanding complex traits and developing curative therapies, the need for large-scale genome editing tools capable of modifying hundreds to thousands of bases is rapidly growing^1,2^. Such capabilities are crucial in diverse fields including synthetic biology^3^, developmental biology^4^, disease modeling^5,6^ and gene therapy^7^, where they support applications such as multigene circuit engineering^8^, reconstruction of regulatory domains^9^, modification of DNA duplications^10^ and rewiring of complex genetic networks underlying human diseases^11^. Traditional large-scale DNA engineering tools, such as recombinases^12^, integrases^13^ and resolvases^14^, are enzyme classes that mediate DNA rearrangement with distinct mechanisms and roles. Recombinases (for example, Cre and Flp) enable precise site-specific modifications^15–17^, integrases (for example, λ and φC31) insert viral DNA into host genomes directionally^18,19^, and resolvases (for example, Tn3 and RuvC) resolve recombination intermediates to maintain genome integrity^20,21^. These tools are foundational in molecular biology, synthetic biology and gene therapy for targeted and dynamic genome editing^22–24^.

**Fig. 1:**
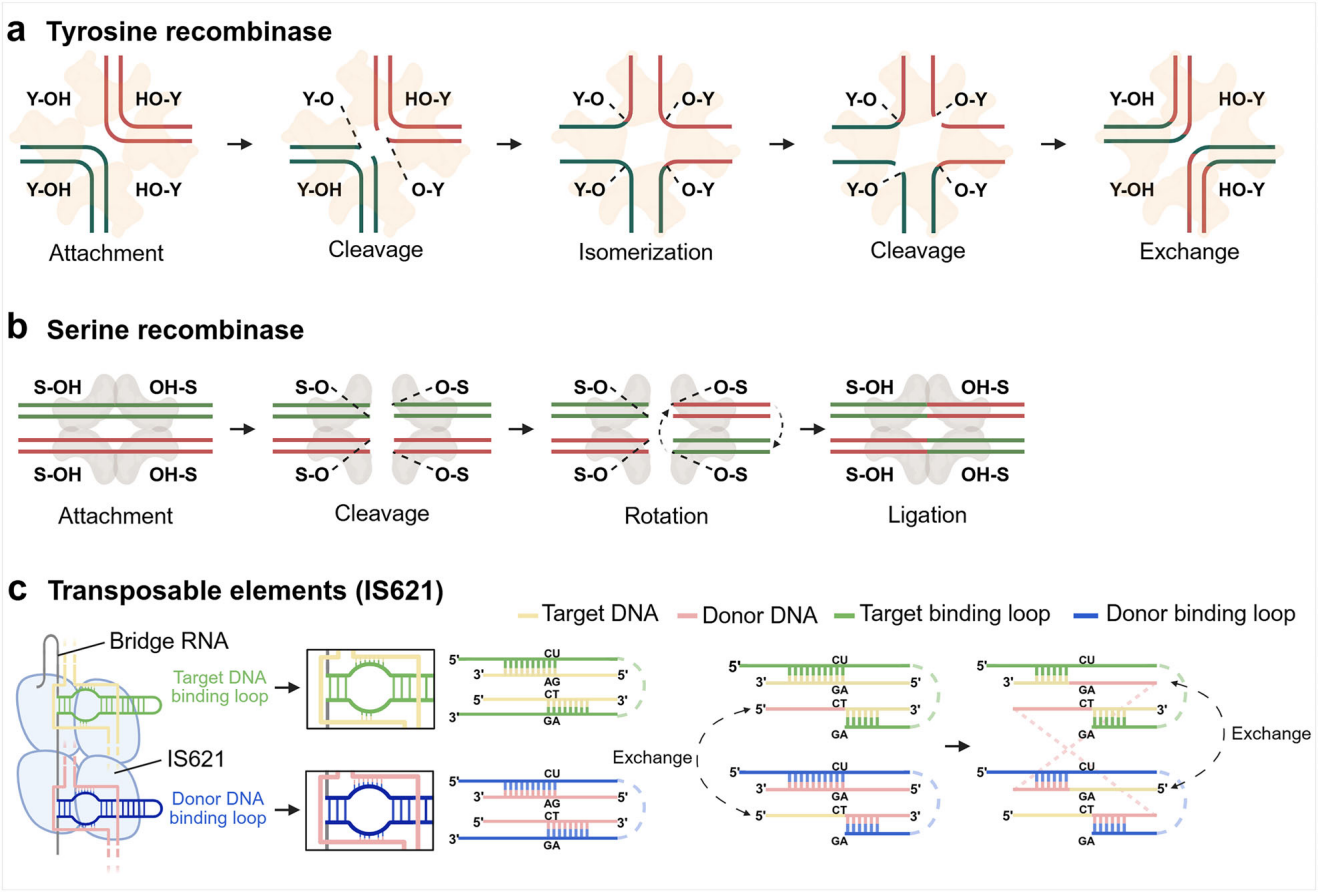
Mechanisms of DNA targeting by recombinase enzymes. **a**, Tyrosine recombinases induce single-strand cuts in both the target and donor DNA, facilitating strand exchange and religation for recombination. The green or red marked curved lines indicate target or donor DNA, Y denotes tyrosine active residue in tyrosine recombinases and OH represents the hydroxyl residue in target or donor DNA. **b**, Serine recombinases create double-strand breaks in the target and donor DNA. Through domain rotation within the recombinase, cleaved DNA strands are rotated and religated, enabling DNA recombination. The green or red marked lines indicate target or donor DNA, S denotes serine active residue in serine recombinases and OH represents the hydroxyl residue in target or donor DNA. **c**, The transposable element, IS621 recognizes its target using a complementary bridge RNA without requiring a landing pad sequence. It induces DNA cleavage and mediates recombination in a manner similar to tyrosine recombinases, involving single-strand cuts, strand exchange and religation. The yellow and pink lines represent the target DNA and donor DNA, and the green and blue stemloops represent the target binding loop and donor binding loop of bridge RNA, respectively.

The Cre-lox system, a prototypical tyrosine recombinase derived from bacteriophage P1, has become one of the most extensively utilized tools for precise genome engineering in eukaryotic and mammalian systems^25–28^(Fig. 1a and Table 1). By contrast, serine recombinases—such as Bxb1 integrase, phiC31 integrase and transposases—offer irreversible recombination with simpler mechanisms and higher efficiency across a broad range of cell types^29–32^(Fig. 1b and Table 2). These enzymes have driven advancements in genetic engineering in both prokaryotic and eukaryotic hosts^33–35^, enabling the development of transgenic models, including *Drosophila*^36^, zebrafish^37^, medaka fish^38^, mouse^39^, chickens^40^ and pigs^41,42^. In addition to these recombinases, which have limited target recognition, in silico approaches have identified recombinase-like sequences with novel properties^43^. Notably, IS110 family elements (IS621) demonstrate target-specific gene recombination capacity through reprogrammable bridge RNAs^44,45^ (Fig. 1c). While traditional site-specific recombination systems have enabled precise DNA rearrangements, their editing capacity remains limited, largely constrained by dependence on predefined recognition sequences, relatively small target regions and low efficiency^22,46^. As a result, more versatile and scalable platforms are required to meet the demands of modern genome engineering.

**Table 1:** Cre and other site-specific tyrosine recombinases.

Recombinase module	Source organism	Function	PDB ID	References
Cre	Bacteriophage P1	Genetic excision, inversion and translocation	1NZB	^150^
XerA, XerC, XerD and XerH	XerA: *Thermoplasma acidophilum DSM 1728* XerC: *Pyrococcus abyssi* XerD: *Escherichia coli* XerH: *Helicobacter pylori 26695*	Excision: dimer reduction in the E. coli chromosome as well as in many other bacterial chromosomes and some plasmids	XerA: 5HXY XerC: 4A8E XerD: 1A0P XerH: 5JK0	XerA^151^ XerC − XerD^152^ XerH^153^
XisA and XisC	*Enterococcus faecalis*	Excision: for developmentally regulated gene activation in Anabaena	1Y6U	^154^

Each recombinase is listed with its source organism, primary function, corresponding PDB (https://www.rcsb.org/) deposit ID (if available) and relevant references. A dash indicates that the information is not available or not applicable.

**Table 2:** BxbI and other site-specific serine recombinases.

Recombinase module	Source organism	Function	PDB ID	Reference
BxbI	*Mycobacterium smegmatis*	Phage BxbI integrase	−	−
A118	*Listeria*	Phage A118 integrase	4KIS	^155^
phiC31	*Streptomyces*	Phage phiC31 integrase	4BQQ	−
TP901	*Lactococcus*	Phage TP901-1 integrase	3BVP	^156^
phiRV1	*Mycobacterium tuberculosis*	Prophage-like element integrase	6LG3	^157^
TnpX	*Clostridium*	Integrative mobile element Tn4451 transposase	2MHC	^158^

Each recombinase is listed with its source organism, primary function, corresponding PDB (https://www.rcsb.org/) deposit ID (if available) and relevant references. A dash indicates that the information is not available or not applicable.

## Strategies for large-scale DNA engineering using existing recombinases

**Fig. 2:**
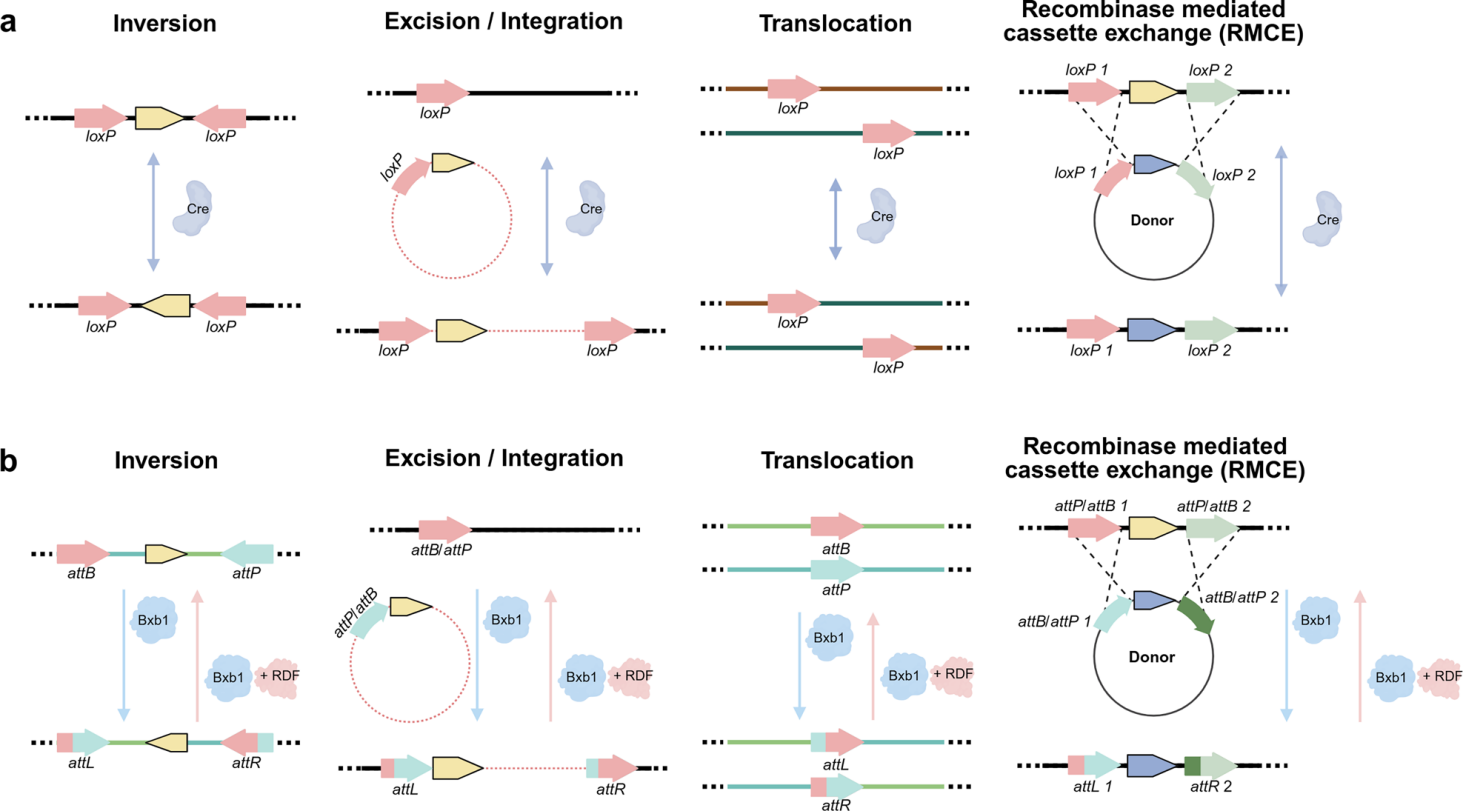
Large-scale DNA editing using recombinase enzymes. **a**, Tyrosine recombinases (representative enzyme, Cre) require recognition of short sequences such as loxP (recognition sequence for Cre recombinase) for target DNA and donor DNA recombination. These sequences, with palindromic structures, remain unchanged after recombination. Depending on the orientation and location of the recognition sites, tyrosine recombinases can perform inversions, excisions, integrations, translocations and cassette exchanges. loxP1 and loxP2 represent different types of loxP recognition sequence. **b**, Serine recombinases (representative enzyme, BxbI) recognize *attB/attP* sequences in the target or donor DNA, respectively. Following unidirectional recombination, the hybrid sequences attL and attR are formed. Similar to tyrosine recombinases, serine recombinases enable large-scale DNA editing, including inversions, excisions, integrations, translocations and cassette exchanges, based on the orientation and location of recognition sequences (*attB/attP*). RDF, recombination directionality factor; RMCE, recombinase-mediated cassette exchange.

Site-specific recombinations have been widely employed for efficient genetic manipulation^47^, enabling the insertion, excision and exchange of target genes across diverse cellular and tissue contexts^17,48^(Fig. 2). Key technologies include Recombinase-Mediated Cassette Exchange (RMCE)^49^, Serine and Tyrosine Recombinase-Assisted Integration of Genes for High-Throughput Investigation (STRAIGHT-IN)^46^, Synthetic Chromosome Rearrangement and Modification by LoxPsym-mediated evolution (SCRaMbLE)^50,51^, Serine Integrase Recombinational Assembly (SIRA)^52^, Dual Integrase Cassette Exchange (DICE)^53^, Serine recombinase–Assisted Genome Engineering (SAGE)^54^ and Recombinase-Mediated Twin Site Targeting (RMTT) technologies^55^ based on tyrosine (Fig. 2a) or serine (Fig. 2b) recombinases. However, the reliance on DNA recognition site ‘landing pad sequence’ for recombinase function in the mammalian cell environment limits their wide application in biomedical and therapeutic research^43^. Traditional recombination-based approaches, such as the Cre-lox system, exhibit site specificity but lack programmability and guide RNA dependency. Despite their widespread application, including in in vivo studies, there remains a critical need to develop next-generation technologies that are programmable and guided by RNA, thereby enabling more precise and flexible genome engineering.

## Advances in target-specific DNA modification using RNA-guided CRISPR system

**Fig. 3:**
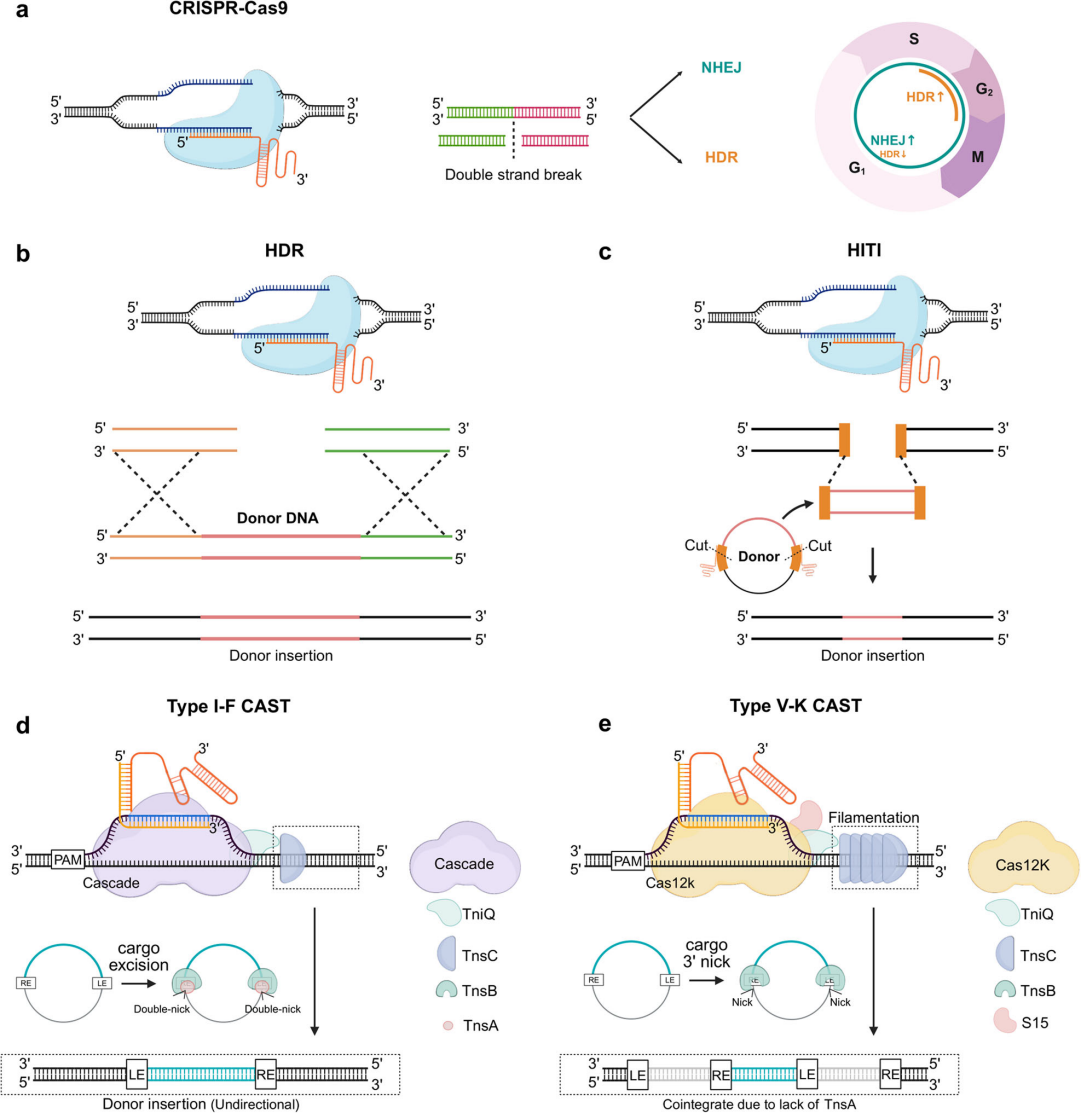
Large-scale DNA engineering strategies utilizing CRISPR systems, either through the induction of double-strand breaks or via DNA break-free approaches. **a**, The CRISPR–Cas9 system induces double-strand breaks in target DNA using reprogrammed guide RNAs, enabling effective genome editing through DNA repair pathways such as NHEJ or homology-directed repair (HDR). The activation of each DNA repair pathway is intricately regulated in a cell-cycle-dependent manner, with distinct mechanisms predominating at specific phases. **b**, HDR enables high-fidelity integration of donor DNA by leveraging homology arms that are complementary to the genomic regions flanking the target locus, facilitating sequence-specific recombination. **c**, Homology-independent targeted integration (HITI) achieves targeted donor DNA insertion through the NHEJ pathway. The donor template is designed to contain sequences identical to the endogenous cleavage site, thereby enabling orientation-specific and precise integration. **d**, Type I-F CRISPR-associated transposase (CAST) enables double-strand break-free genomic integration via a multisubunit cascade complex that recognizes the target DNA through a CRISPR RNA (crRNA). Upon target engagement, the ATP-dependent recruitment of TnsC facilitates the bridging of the cascade complex to the transposition machinery. The target site of the donor DNA is accurately recognized and precisely cleaved by TnsA and TnsB, thereby allowing unidirectional integration at the target locus through a single TnsC-dependent mechanism. **e**, Type V-K CAST utilizes a single-effector Cas12k protein to mediate crRNA-guided target recognition. In the absence of TnsA, complete excision of the donor DNA is precluded, resulting in integration through a cointegration mechanism. TnsC assembles into ATP-dependent filaments along the DNA substrate, promoting accurate target site selection and stabilizing the integration process. LE, left end; RE, right end. The figures were created using BioRender software (BioRender.com).

To address the limitations of existing recombinases, DNA modification technologies are being developed such that they can be reprogrammed to target DNA using target-specific DNA binding modules^56^. Among these, the clustered regularly interspaced short palindromic repeats (CRISPR) system is the most widely used owing to its accuracy and the ease of fabricating guide RNAs to match target DNA^57–59^ (Fig. 3). CRISPR systems are classified into class I (types I, III and IV), which require multiple Cas components, and class II (types II, V and VI), which use a single Cas enzyme. These CRISPR systems require a mature CRISPR RNA and trans-activating RNA for target recognition and cleavage^58^. To facilitate genome editing, RNA-guided CRISPR effectors are employed to induce DNA double-strand breaks (DSBs) at specific sites, leveraging DNA repair mechanisms to copy donor DNA sequences into the target genome^60,61^ (Fig. 3a–c). To enhance homology-directed repair (HDR) efficiency (Fig. 3b), strategies have been developed, including engineering CRISPR effectors, optimizing donor DNA design and suppressing the non-homologous end-joining (NHEJ) pathway using small molecules, enabling the knock-in of large DNA fragments into target genes^62–69^. The HDR depends on cell cycle, it is only positive in S or G2 phase and also generate indel mutations caused by DNA double-strand breaks and following the NHEJ pathway. Alternatively, technologies such as homology-independent targeted insertion (HITI) have emerged (Fig. 3c), which simultaneously induce DSBs at endogenous sites within cells and in donor DNA, facilitating DNA fragment insertion via the NHEJ pathway^70,71^. The HITI method does not only depend on the cell cycle but also generates indel mutations dominantly, which is a critical limitation. Despite their efficacy, these methods rely on the DSB-inducing capabilities of CRISPR effectors, which inherently carry challenges such as off-target effects and unintended DNA modifications at on-target sites^72^. Recent advancements have focused on using modified forms of CRISPR–Cas effectors, such as nickases or inactivated versions, to enhance homology-directed repair and enable more precise DNA insertion while minimizing associated risks^73,74^. Recently, on the other hand, gene editing using transposon systems associated with CRISPR has also been reported (Fig. 3d,e and Table 3). In 2017, Peters et al. uncovered CRISPR elements work with Tn7-like transposons^75^. CASTs are RNA-guided elements that integrate into DNA by base-pairing target protospacers with complementary CRISPR RNA spacers and recognizing protospacer adjacent motifs (PAMs)^75,76^. In additoin, CAST systems utilize the conserved DDE-family transposase TnsB, which catalyzes strand transfer during transposition, together with accessory factors TnsC and TniQ^77^. These systems have been identified and developed from diverse bacterial strains^78,79^. So far, several CAST subtypes—namely I-C, I-D, I-E, I-F, IV-A and V-K—have been identified, among which subtypes I-F and V-K are the most well-characterized^80–83^. The type I-F CAST system encodes Cas6, Cas7 and Cas8, which together form the Cascade complex, and facilitates efficient insertion of genetic elements by employing an RNA-guided DNA cut-and-paste transposition mechanism^78,84,85^(Fig. 3d). The cascade and guide RNA, together with TniQ which recruit TnsC, directs target DNA recognition, while TnsA, TnsB and TnsC form a heteromeric transposase complex that catalyzes DNA cleavage and transposition^78,86^. DNA integration by type I-F CASTs occurs approximately ~50 bp downstream of the target site^85^. In type V-K CAST systems (Fig. 3e), transposition is orchestrated by the single-effector protein Cas12k, with DNA integration occurring 60–66 base pairs downstream of the PAM site^79^. Unlike the type I-F CAST system, the type V-K CAST mechanism generates cointegrate products through a replicative pathway, owing to the absence of the endonuclease TnsA^85,87^. To enable successful DNA transposition, the TniQ factor and ribosomal protein S15 are additionally recruited^88^, promoting the TniQ-mediated assembly of TnsC into a filamentous structure along the target DNA. This arrangement facilitates subsequent interaction with TnsB, thereby culminating in the formation of the complete integration complex^89^.

**Table 3:** CRISPR-based large-scale DNA modification system.

Name	Recombinase system (mutant type)	Deleted or substituted gene size (bp), efficiency (%)	Inserted gene size (bp), efficiency (%)	Delivery method (target organism)	Reference
CAST (type I-F)	PseCAST	–	~1.3 kb, ~ 1% (cell line)	Lipofection (HEK293T cell lines)	^101^
CAST (type V-K)	MG64-1	–	~3.6 kb, < 1% (cell line)	Lipofection (K562, HEK293T cell lines)	^102^
evoCAST (type I-F)	Evolved PseCAST: TnsA (P88T, I147V, V170L, F180L, F182L), TnsB (F43S, Y349N, P352T, A390V, D396N, Q410K, H464R, V526E, Q549R, Q594L), TnsC (R197I, N314K), Cas7 (A347K) and Cas8 (N125D, A244N, A410R)	–	~ 4.7 kb, ~15% (cell line)	Lipofection (HEK293T, HeLa, HuH7 cell line)	^103^
PRIME-Del	–	Deletion, ~10 kb, 1-30% (cell line)	–	Lipofection or Lentiviral (HEK293T cell line)	^121^
TJ-PE	–	Substitution, 74 bp, average 2.5% (mice)	~500 bp, ~ 11.4% (cell line)	Lipofection (HEK293T cell line, *Fah* mutant B6/Tyr- mice)	^120^
Twin-prime editing (twinPE)	–	Deletion ~818 bp Inversion ~40 kb (cell line)	Maximum size 113 bp, ~10% (cell line)	Lipofection (HEK293T cell line)	^118^
PASTE	WT Bxb1	–	~36 kb	AAV or AdV delivery	^126^
PASSIGE	WT Bxb1	–	~3 kb	lipofection	^127^
evoPASSIGE	evoBxb1 (V74A)	–	~10.5 kb	lipofection	^127^
eePASSIGE	eeBxb1 (V74A, E229K and V375I)	–	~10.5 kb	lipofection	^127^
PE (with hyperactive integrase)	Hyperactive PhiC31	–	~6.6 kb	lipofection, electroporation	^128^
PE (with hyperactive integrase)	Hyperactive Bxb1 (I87L, H95Y, V122M, A369P and E434G)	–	~15.7 kb	lipofection, electroporation	^128^

Each CRISPR-based technology is listed according to the recombinase system utilized, the type of editing outcome (deletion, substitution or insertion), editing efficiency, target organism and the corresponding reference. A dash indicates that the information is not available or not applicable.

CAST systems present a unique strategy for integrating large genetic elements into specific genomic loci without introducing double-strand breaks, relying solely on guide RNA for target recognition. In various prokaryotic hosts, this method has demonstrated nearly complete insertion in *Escherichia* *coli*, enabling the stable integration of donor sequences up to approximately 15.4 kb with type I-F CAST^90–95^ and as much as 30 kb using type V-K variants^79,96–100^. Despite these successes, applications in mammalian cells are still in early stages (Table 3). Type I-F CAST, for example, has only achieved about 1% editing efficiency in HEK293 cell line with approximately 1.3 kb sized donor DNA^101^. As another example, to facilitate large-scale DNA editing in human-derived cells, a V-K CAST variant incorporating a fusion protein (nAnil-TnsB) was codelivered with a 2.6 kb donor DNA containing the nAnil recognition sequence^99^. As a result, a large-scale DNA editing efficiency of up to 0.06% was observed in HEK293T cells targeting plasmid DNA. Fortunately, substantial progress has been made through various biological approaches, yielding promising results for the future application of CAST systems in humans. Notably, the V-K CAST system MG64-1 was identified via metagenomic mining^102^. In HEK293 cells, this system achieved approximately 3% integration efficiency of a 3.2 kb donor at the AAVS1 locus, and integration rates of ~3% and <0.05% for a 3.6 kb therapeutic donor in K562 and Hep3B cells, respectively. More recently, a PseCAST system engineered through directed evolution has exhibited considerable potential for future use in complex biological contexts^103^.

## Recent advances in prime editing technology and its application in large-scale DNA modification

**Fig. 4:**
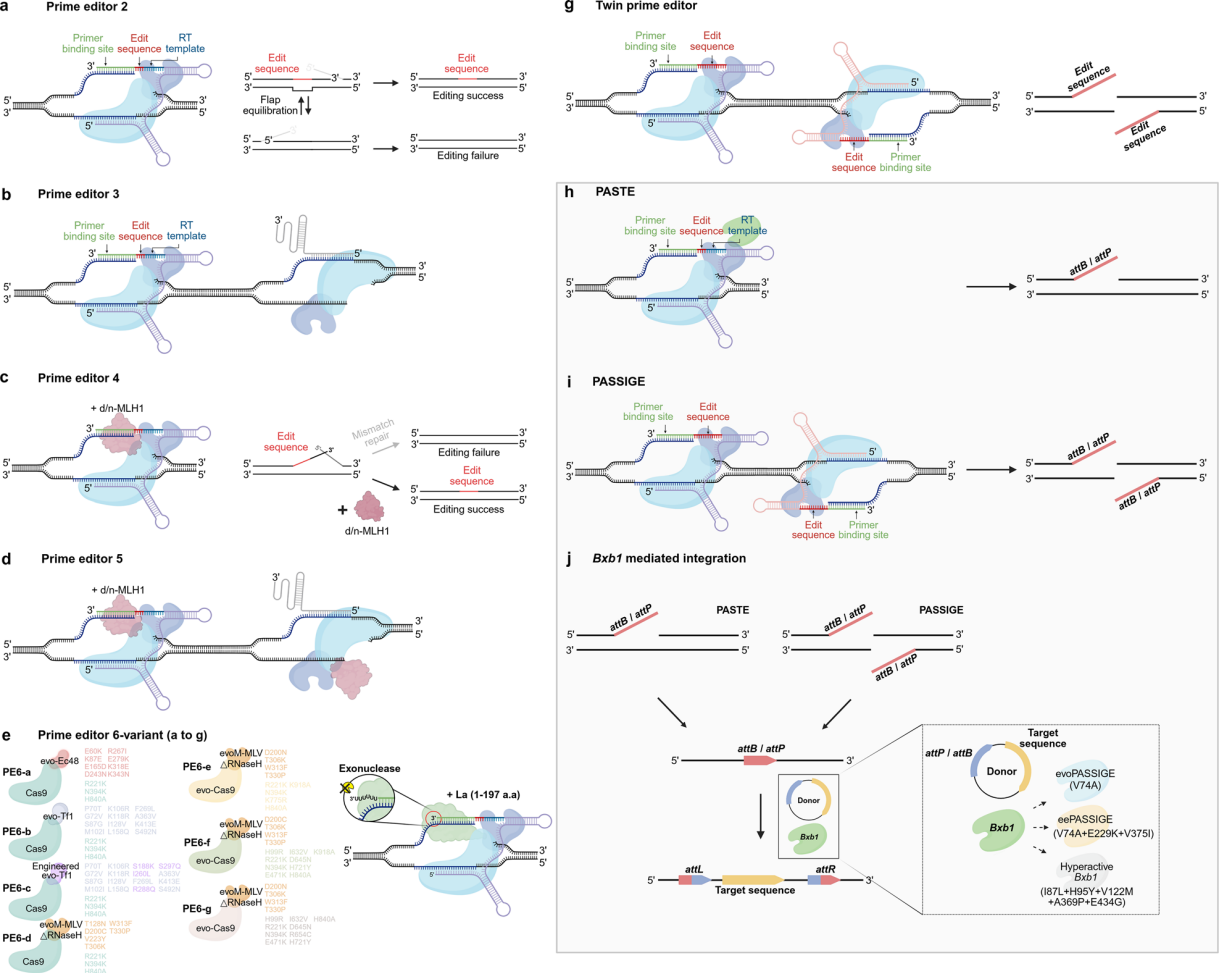
Large-scale DNA editing using prime editing-based technologies. **a**, Prime editing employs CRISPR nickases to introduce nicks instead of double-strand breaks. An RT copies pegRNA information into the target DNA, enabling precise deletions, insertions and substitutions. PE2 utilizes pegRNA and a single CRISPR nickase-RT system. The extended 3′-flap DNA, derived from the pegRNA template, undergoes an equilibrium state of association and dissociation with the target DNA, thereby enabling the induction of the intended gene mutation through mismatch repair (MMR) mechanisms. **b**, PE3 enhances editing efficiency by introducing an additional nick opposite the non-target strand nick of the PE to control DNA substitution mediated by base excision repair. **c**,**d**, PE4 (**c**) and PE5 (**d**) improved PE2 and PE3, respectively, by adding functional domains to the prime editor (PE). Using a dominant-negative MLH1 (d/n-MLH1) protein disrupts the MMR complex, preventing MMR repair and enhancing desired sequence insertion. **e**, PE6 integrates a compact, optimized RT into the PE, improving editing efficiency in a smaller construct. **f**, PE7 uses the human-derived La protein to stabilize pegRNA by protecting its 3′-end tail from exonuclease degradation, enhancing editing efficiency. **g**, Twin-prime editing employs two PEs to form complementary strands via RT synthesis, enabling precise insertion, deletion and substitution through base pairing. **h–j**, Programmable DNA modification strategies leveraging site-specific recombinases in conjunction with prime editing technology enable efficient and scalable genome engineering: the PASTE system combines recombinase with a single PE to insert recombinase recognition sequences into target DNA and subsequently facilitate donor DNA recombination (**h**); the PASSIGE system utilizes twin-prime editing to insert recombinase recognition sequences into the target DNA (**i**); and sequential donor DNA recombination is achieved using site-specific recombinase (BxbI) (**j**). The BxbI recombinase employed in this system has an enhanced version developed through directed evolution to improve recombination efficiency. d/n-MLH1, dominant-negative MLH1 protein; M-MLV, Moloney murine leukemia virus. The figures were created using BioRender software (BioRender.com).

Prime editing is a technological advancement designed to enable the precise insertion of desired genes into target DNA in living organisms using a CRISPR-based approach^58,104–106^(Fig. 4a). This technology links a CRISPR–Cas9 module to a reverse transcriptase (RT) enzyme^107^, enabling targeted DNA editing—such as base modifications, including insertions, deletions and substitutions of up to a few base pairs—guided by reprogrammed prime editing guide RNA (pegRNA)^108–110^. Since its first description in the year 2019^104^, prime editors have been continuously developed and optimized to increase editing efficiency^111^. For example, prime editor version 2 (PE2) version demonstrates precise editing in human-derived cell lines without inducing DSBs^112^. To enhance the incorporation of edits, the PE3 version introduces an additional nick in the target strand, promoting base excision repair (Fig. 4b). However, the additional nicking induced in the target strand can result in inefficiencies in various human-derived cell targets and induce unintended modifications, including insertions and deletions^104,113,114^. To address these issues, the PE4 and PE5 systems were developed, which significantly improve prime editing efficiency by combining PE2 and PE3 system with transient expression of a dominant-negative form of MLH1, which is a mismatch repair-related protein^115^ (Fig. 4c,d). Further advancements, such as the PE6a, PE6b and PE6c systems, use mutated evo-Ec48 and evo-Tf1 as RTs^116^(Fig. 4e). Moreover, PE6d incorporates RNaseH-truncated and engineered Moloney murine leukemia virus (M-MLV) RTs. Further mutations to the CRISPR–Cas9 module have led to the development of PE6e-g, showing increased editing efficiency^116^. Integrating a small RNA-binding La protein to generate PE7 enhanced prime editing efficiency by up to 21.2-fold while reducing on-target insertions and deletions^117^(Fig. 4f). The endogenous La protein binds to poly-uridine tracts in RNAs, protecting them from exonuclease degradation of 3′ end of pegRNA′. Notably, multiple paired prime editing strategies (Fig. 4g and Table 3), including twin-prime editing (twinPE)^118^, bi-direction prime editing (Bi-PE)^119^, template-jumping prime editing (TJ-PE, sequential utilization of two pegRNAs enables the linkage of two distal genomic loci, facilitating insertions or exon replacement)^120^ and PRIME-Del (the intervening sequence is completely removed, followed by seamless ligation of the flanking homology arms)^121^, have been developed to enable the simultaneous editing of both DNA strands, thereby enhancing the overall efficiency and precision of large-scale DNA modifications, such as deletion, insertion and substitution in target cell lines. Another technique that improves the efficiency of prime editing involves the engineering of prime editing components, including nickases and RTs^122^, which can be conjugated together or split depending on the delivery vehicles such as adeno-associated virus (AAV)^123–125^. Programmable addition via site-specific targeting elements (PASTE) technology is an optimized method for large gene insertion via prime editing^126^ (Fig. 4h,j and Table 3). This approach enables large gene incorporation with high target specificity, and minimal double-strand breaks, by sequentially activating target site priming via prime editors and donor DNA insertion mediated by site-specific recombinases. PASTE technology utilizes the same components as prime editing, with the addition of Bxb1 integrase tethered to M-MLV RT. Moreover, the pegRNA of prime editing is extended with a landing pad (*attB* sequence) for Bxb1 recognition^126–128^. In this case, PASTE efficiency depends on target loci and pegRNA parameters (length of PBS, RT and *attB* sequences). According to the findings validated so far, the PASTE system allows the directed integration of large sequences (~36 kb) to target sites using serine integrase^126–128^.

Following the recent development of PASTE technology, which integrates recombinases with prime editing, various studies have focused on improving large gene insertion efficiency by engineering recombinase enzymes^127,128^. One such advancement, prime-assisted site-specific integrase gene editing (PASSIGE) technology, uses twin-prime editing to insert attP/B sites for Bxb1 binding, followed by foreign DNA insertion into the target using the highly efficient Bxb1 recombinase (Bxb1 is either coexpressed with the prime editor via a self-cleaving 2A peptide within the same expression construct or individually overexpressed)^127^ (Fig. 4i,j and Table 3). Evolving Bxb1 through phage-assisted continuous evolution (PACE) and phage-assisted non-continuous evolution (PANCE) introduced mutations, resulting in evolutionary PASSIGE (evoPASSIGE) and enhanced evolutionary PASSIGE (eePASSIGE), which showed 9.1-fold and 16-fold improvements in integration efficiency over the PASTE system, respectively (Fig. 4j, inset). In addition, a similar approach revealed recombinase variants that enhanced large gene insertion efficiency^128^. Hyperactive versions of Bxb1 recombinases containing specific mutations demonstrated a 3.1-fold increase compared with the wild-type recombinase.

## Off-target, byproduct and translocation issues associated with large gene insertion technology

The CRISPR system recognizes target DNA based on guide RNA; however, its inherent tolerance for mismatched bases between the guide RNA and DNA can lead to off-target effects. These off-target CRISPR malfunctions have been reported to cause chromosomal breaks, translocations and associated developmental defects, neurodegeneration, immunodeficiency, infertility and increased cancer susceptibility^129–131^. Similarly, prime editing technologies for large gene insertion are also susceptible to off-target activities owing to guide RNA-based target recognition^126–128^. Several methods have been developed to detect the off-target effects of prime editing^132,133^, including nDigenome-seq, which uses nCas9 H840A to generate single-strand breaks in the non-target site for off-target screening^132^. In addition, PE-tag, a cell-based assay, was developed recently to analyze prime editing off-target, showing lower off-target editing where the prime editor can operate compared with conventional Cas9 systems^133^. To minimize off-target effects, strategies to improve the target specificity of SpCas9, such as SpCas9-HF1, eSpCas9, HeFSpCas9, EvoSpCas9 and HypaCas9, can be applied^134–138^. When using prime editing for large gene insertion, off-target effects can also occur through recombination by the BxBI enzyme, which allows recognition of the pseudo-*attB* site^127,128,139^. Although current studies report minimal off-target editing effects, further validation studies are necessary to ensure safety.

## Concluding remarks

Recent advances in the discovery and application of CRISPR effectors have enabled targeted gene modifications and large DNA engineering in vivo. However, prime editing, the foundation of current large DNA engineering technologies, still requires significant improvements owing to challenges, such as low gene editing efficiency, narrow targeting scope, unpredictable insertions and deletions and off-target effects caused by guide RNA-based recognition. To overcome these issues, the components of the prime editing, Cas9 nickase (H840A), RT (M-MLV RT) and pegRNA, can be fine-tuned to facilitate target sequence insertion recognized by recombinases. Previous study shows that modifying pegRNA by adding pseudoknots improves its stability, increasing efficiency by three- to fourfold^111^. Alternatively, SpCas9 orthologs can be used as an alternative to prime editing. For example, although *Streptococcus pyogenes* Cas9 (SpCas9) recognizes the 5′-NGG-3′ sequence, engineered nucleases with minimal and flexible PAMs, such as the SpG and SpRY variants, have been developed^140–143^. Prime editors utilizing PAM-flexible Cas9 variants demonstrate expanded targeting ranges, achieving up to 51.7% prime editing activity in HEK293T cells^144^. Prime editing also can be directly induced using orthologs from *Francisella novicida* Cas9, which shows high specificity for target DNA^145^. The use of highly accurate, modular prime editing technologies could enable precise, large-scale genome editing. In addition, the CRISPR–Cas12f effector, known as a micromodule for high-efficiency human cell delivery^146–149^, can also be developed as a miniaturized prime editor for AAV loading. These approaches are capable of inducing high-efficiency gene editing in both plants and animals and hold significant potential for the future development of human-targeted gene therapies. Addressing the large-scale gene editing challenges from multiple perspectives, either by improving the CRISPR components or the delivery methodology, could facilitate accurate and effective gene editing across various biological targets. These advancements offer broad application potential across various fields, particularly within human systems.
