# Author Correction: Advances in large-scale DNA engineering with the CRISPR system

**DOI:** 10.1038/s12276-026-01695-2

**Published:** 2026-03-31

**Authors:** Lee Wha Gwon, Isabel Wen Badon, Youngjeon Lee, Ho-Joong Kim, Seung Hwan Lee

**Affiliations:** 1 National Primate Research Center, Korea Research Institute of Bioscience and Biotechnology, Cheongju, Republic of Korea; 2 KRIBB School of Bioscience, University of Science and Technology, Daejeon, Republic of Korea; 3 Department of Life Science, Chung-Ang University, Seoul 06974, Republic of Korea; 4 Department of Biology and Environmental Science, University of the Philippines Cebu, Cebu City, Philippines; 5 Department of Chemistry, Chosun University, Gwangju, Republic of Korea

**Keywords:** Gene therapy, Molecular engineering

Correction to: *Experimental & Molecular Medicine* 10.1038/s12276-025-01530-0, published online 01 September 2025

After online publication of this article, the authors noticed an error in the Figure image section.

In Fig. 2B, the right end arrow color is incorrect. In Fig. 4, the edit sequence and RT template location of pegRNA, the prime editor component, are incorrect. In addition, the PE6 mutation was misannotated.

The correct figures of this article should have appeared as shown below.

Incorrect Figure 2
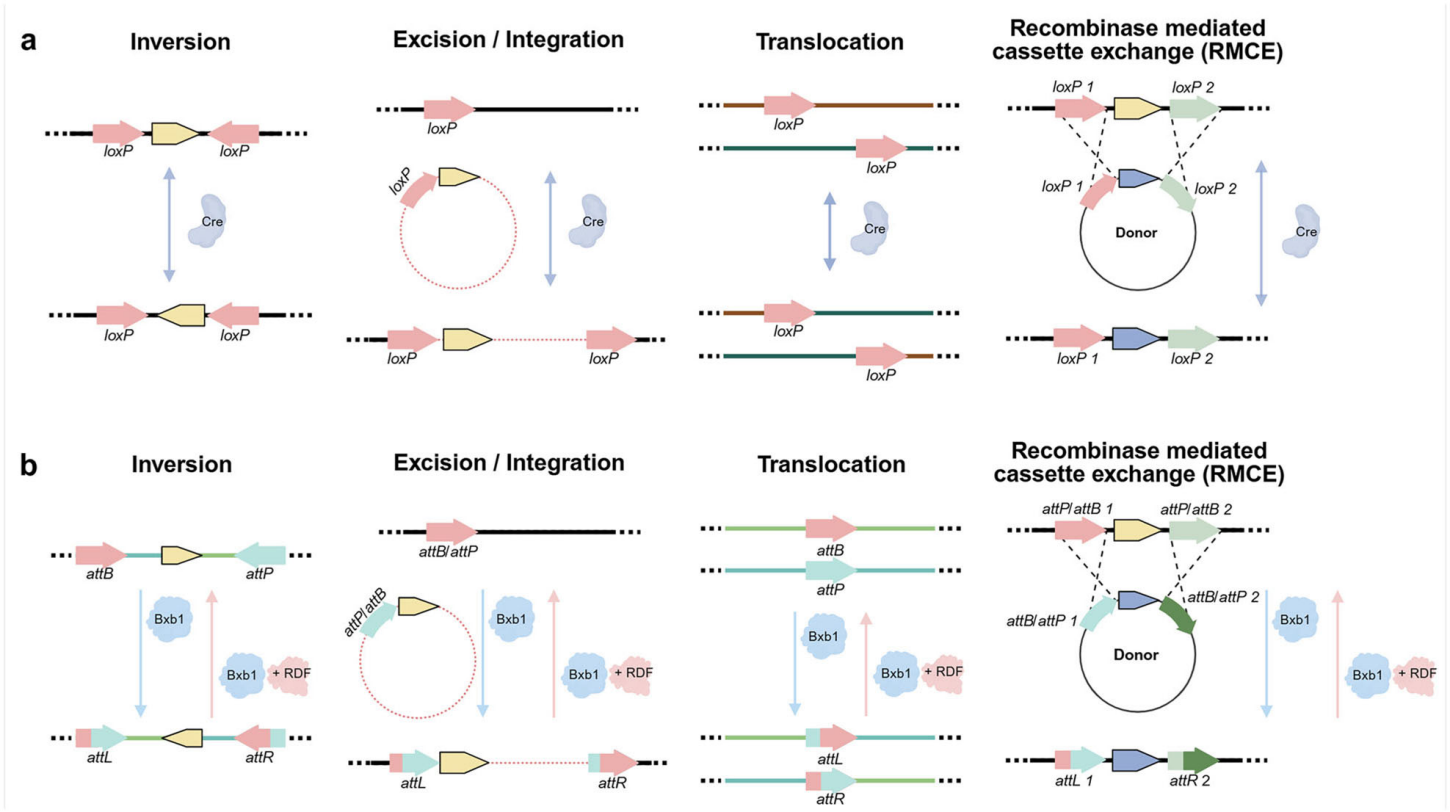


Correct Figure 2
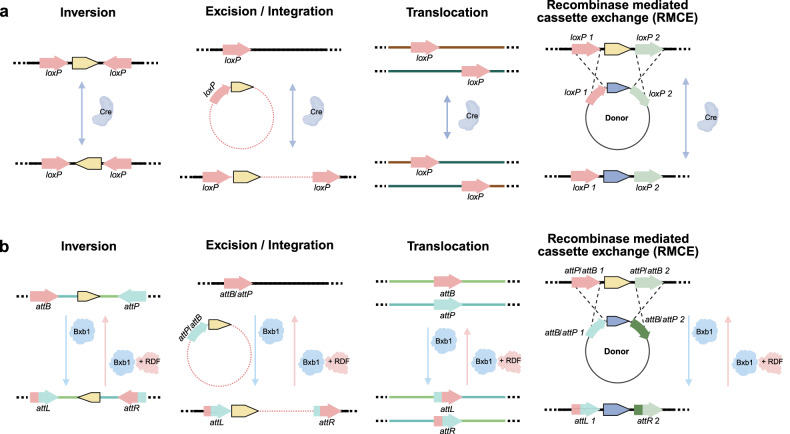


Incorrect Figure 4
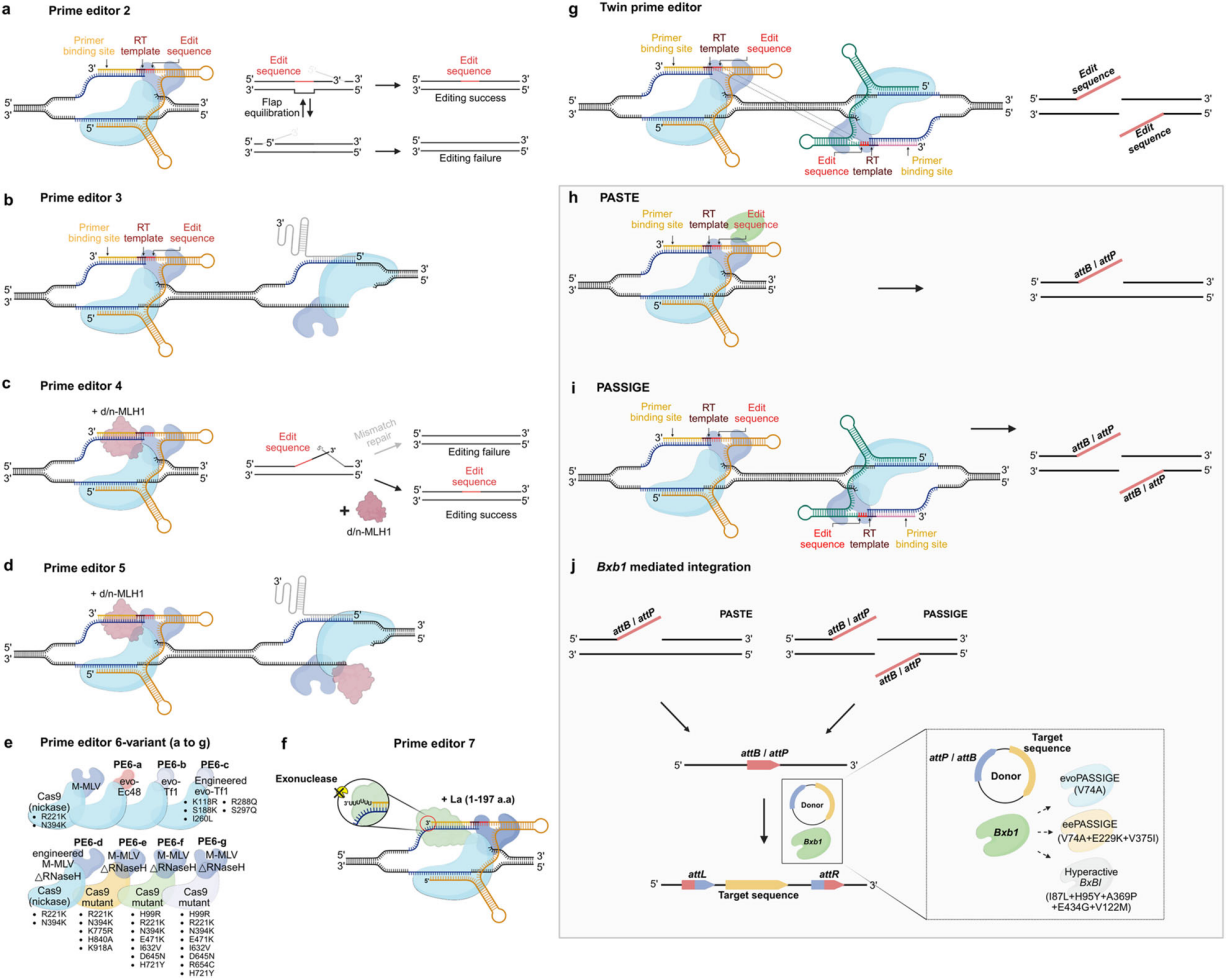


Correct Figure 4
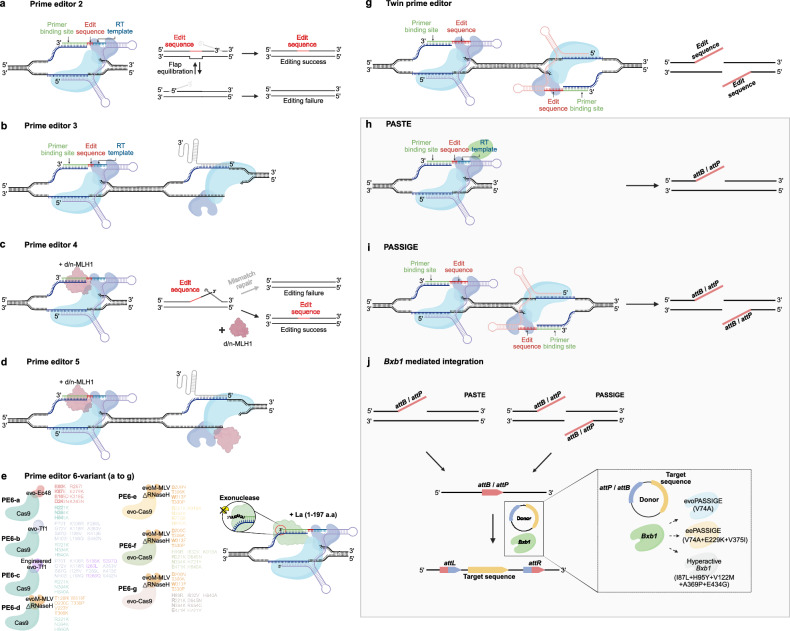


The authors apologize for any inconvenience caused.

The original article has been corrected.

